# RIPK1 Is Cleaved by 3C Protease of Rhinovirus A and B Strains and Minor and Major Groups

**DOI:** 10.3390/v13122402

**Published:** 2021-11-30

**Authors:** Sarah N. Croft, Erin J. Walker, Reena Ghildyal

**Affiliations:** 1 John Curtin School of Medical Research, Australian National University, Canberra 2601, Australia; Sarah.Croft@anu.edu.au; 2 Centre for Research in Therapeutic Solutions, Faculty of Science and Technology, University of Canberra, Canberra 2617, Australia; ejwalker08@gmail.com

**Keywords:** rhinovirus, 3C protease, RIPK1, programmed cell death

## Abstract

Rhinoviruses (RV), like many other viruses, modulate programmed cell death to their own advantage. The viral protease, 3C has an integral role in the modulation, and we have shown that RVA-16 3C protease cleaves Receptor-interacting protein kinase-1 (RIPK1), a key host factor that modulates various cell death and cell survival pathways. In the current study, we have investigated whether this cleavage is conserved across selected RV strains. RIPK1 was cleaved in cells infected with strains representing diversity across phylogenetic groups (A and B) and receptor usage (major and minor groups). The cleavage was abrogated in the presence of the specific 3C protease inhibitor, Rupintrivir. Interestingly, there appears to be involvement of another protease (maybe 2A protease) in RIPK1 cleavage in strains belonging to genotype B. Our data show that 3C protease from diverse RV strains cleaves RIPK1, highlighting the importance of the cleavage to the RV lifecycle.

## 1. Introduction

The efficiency of productive viral infection relies on the ability of a virus to establish infection within a single cell and viruses have evolved capabilities to control the cellular environment to escape the innate immune response, prolong host-cell survival, limit antigen-presentation, promote viral replication, and facilitate virus assembly or release. The execution of apoptosis may result in cleavage of factors required for viral genome replication, translation, autophagy, and virus release, and ultimately cell shutdown, thus preventing viral replication. Programmed cell death (PCD) is a host response to virus infection in order to limit the spread of infection and contain the damage. Viruses have evolved several mechanisms to modulate PCD.

The timely inhibition and then the induction of apoptosis by Picornaviruses probably allows optimal viral replication [1]. The suppression of proapoptotic factors may be a viral strategy to shut down innate immune responses. Importantly, several Picornaviruses have evolved to control cell death signalling by multiple mechanisms, as reviewed in [2].

While apoptosis has been argued as both pro- [3] and anti-viral [4], it has been shown, that in the context of rhinovirus (RV) infection, exogenous induction of apoptosis limits viral replication [5,6]. Additionally, the converse has been shown where treatment with apoptosis inhibitors has resulted in increased RV titre [4,6]. There are multiple RV factors that interact with components of the apoptotic signalling pathways, as reviewed in [2]. We, and other groups, have identified the cleavage of Receptor-interacting protein kinase-1 (RIPK1) by the RV 3C protease (3Cpro) [5,7].

RIPK1 is a decision-making protein that plays a vital role in cell viability during embryogenesis, as well as necroptosis and apoptosis signalling. The induction of each cell-survival or cell-death state is dependent on the cleavage or ubiquitination state of RIPK1. At steady-state, RIPK1 is ubiquitinated by cellular inhibitors of apoptosis (cIAPS) [8]. In this form, RIPK1 promotes NF-kB activation and the expression of Flice-inhibitory protein (FLIP), preventing aberrant caspase 8 activation. In a pro-apoptotic state, de-ubiquintinases remove the ubiquitination, thus halting NF-kB signalling. Concurrently, caspase 8, once active, cleaves RIPK1 at Aspartate 324, releasing the RIPK1 death domain to enhance interactions between TRADD and FADD; further promoting apoptosis [8,9,10]. In the absence of caspase 8, RIPK1 interacts with RIPK3 through the RHIM-homology domains. This interaction leads to necroptosis when the heterodimer interacts with mixed-lineage kinase domain-like (MLKL) to oligomerize and form pores within the cellular membrane [11,12,13]. Significantly, RIPK1 knock-out is perinatally lethal in mice, with mice succumbing to systemic inflammation [14]. Recently, human biallelic RIPK1 deficiencies have been described in patients with combined immunodeficiency and paediatric inflammatory bowel disease [15,16]. Interestingly, poly (I:C), a synthetic viral RNA analogue, does not induce apoptosis in cells from these patients.

There are multiple cell fates that could be modulated when RIPK1 is altered and we, and others, have shown that cleavage of RIPK1 by RVA-16 3Cpro [5,7], inhibits apoptosis. We sought to understand whether this was unique to RVA-16, or whether this may be a mechanism of controlling the cellular environment shared by multiple RV serotypes. To this end, we investigated whether 3Cpro cleavage of RIPK1 is conserved across phylogenetic relationships (species A or B) and receptor usage (minor or major groups) within RVs.

## 2. Materials and Methods

### 2.1. Cells

Ohio-HeLa (O-HeLa) cells (ECACC: 84121901) were used for all infection experiments. Cells were maintained in Dulbecco’s modified eagle medium (DMEM) supplemented with 10% Foetal Bovine Serum (FBS) and penicillin/streptomycin/neomycin (PSN).

### 2.2. Viruses

The following major group RVs were used throughout this study: RVA-16 was a gift from E. Dick and W. Busse (Madison, WI, USA), RVB-14 (ATCC: ATCVR284), RVB-83 (ATCC: ATCVR1193). The following minor group RVs were used throughout this study: RVA-2 (Biota Holdings, Melbourne, Australia), RVA-1B (ATCC: ATCVR1645).

### 2.3. Infections and Cell Treatments

All infection experiments were conducted in DMEM supplemented with 2% FBS and PSN. Virus stocks were grown as described [17]. For all infection experiments, the viral inoculum was added to subconfluent monolayers of cells at a multiplicity of infection (M.O.I.) of three. Cultures were rocked occasionally for 1 h before inoculum was removed and replaced with DMEM-2% FBS for times indicated in the text. At 3 h post-infection (hpi) cells were treated with ActinomycinD (ActoD) at 5 µg/mL or remained untreated. At 6 hpi cells were treated with Rupintrivir (Rup.) at 1 mM or remained untreated. At indicated timepoints, cells were processed for assays described below. The timing and dose of ActoD were optimized as previously, to specifically cause apoptosis [5]. The timing and dose of Rup were optimized as previously [5].

### 2.4. Western Blot

At indicated timepoint, cells were washed once with cold phosphate-buffered saline (PBS) and incubated in cold RIPA buffer (150 mM NaCl, 1% Triton X, 0.5% Sodium deoxycholate, 0.1% SDS, 50 mM Tris-HCL) with protease and phosphatase inhibitors (Roche, Basel, Switzerland) for 30 min with rocking. Cell lysates were centrifuged at 12,000 rpm for 10 min to pellet cell debris. The supernatant was collected, and Laemmli Buffer was added before heating at 90 °C for 5 min. Proteins were separated by SDS-Polyacrylamide gel electrophoresis on 8% or 12.5% polyacrylamide gels. Proteins were transferred to a nitrocellulose membrane in Tris-Glycine-ethanol buffer (25 mM Tris, 192 mM Glycine, 20% Ethanol). Non-specific protein binding sites were blocked with 4% Skim milk in PBS before overnight incubation in primary antibody diluted in 1% skim milk in PBST (PBS with 0.1% Tween 20). Proteins separated on 8% gels were probed for eIF4G and PABP while proteins separated on 12.5% gels were probed for RIPK1 and Caspase 3. Blots were then washed in PBST before 2-h incubation in secondary antibody diluted in 1% skim milk in PBST and washing. Enhanced Chemiluminescence (ECL; Perkin Elmer, Waltham, MA, USA) was used to detect bound antibodies. Primary antibodies used throughout this study are as follows; rabbit anti-human RIPK1 (Cell Signalling #D94C12, diluted 1:1000), rabbit anti-human PABP (Cell Signalling #4992, diluted 1:1000), rabbit-anti eIF4G (Santa Cruz #sc11373, diluted 1:1000), rabbit anti-human α/β Tubulin (Cell Signalling #2148S, diluted 1:1000).

### 2.5. Image Analysis

Western blot images were acquired on LiCor Odyssey Fc and digital images were analysed with ImageJ. Profile of each lane was obtained using the Plot Profiles tool. Band intensities were measured and normalized to the corresponding tubulin blot. Microsoft Excel to calculate mean and standard error for the data so obtained.

### 2.6. Viral Replication Assays

At indicated timepoints, infection cultures were frozen at −80 °C. The virus was clarified from cellular debris, followed by endpoint titration as previously described [5].

### 2.7. Flow Cytometry

Infected cells were centrifuged and resuspended in Annexin V binding buffer (10 mM HEPES, 140 mM NaCl, 2.5 mM CaCl_2_). Cells were stained with Annexin V (anti-human Phosphatidylserine antibody)-FITC (ThermoFisher #A13199) and Propidium iodide (Pi) (BD: 556463) before flow cytometric analysis. Data were acquired with BD LSR-II cytometer and FACS-Diva 8.0.1. software. Data analysis was conducted with FlowJo software. Cells were sequentially gated on FSC-A × SSC-A (intact cells), FSC-H × FSC-W and SSC-H × SSC-W (doublet discrimination), Pi × FITC (quadrants for apoptotic and/or necrotic state).

### 2.8. Statistical Analysis

GraphPad Prism 6 was used for all statistical tests. Experiments were repeated three times except for the Flow Cytometry data which represent two independent experiments. For pairwise comparisons of less than three groups, a two-tailed *t*-test assuming equal variance of standard deviation was used to ascertain significance. For multiple comparisons, ordinary-one or two-way ANOVA was used with Tukey’s multiple comparison post hoc test. Statistical significance was accepted at *p* < 0.05.

## 3. Results

### 3.1. Infection with All RV Strains Tested Results in RIPK1 Cleavage

**Figure 1:**
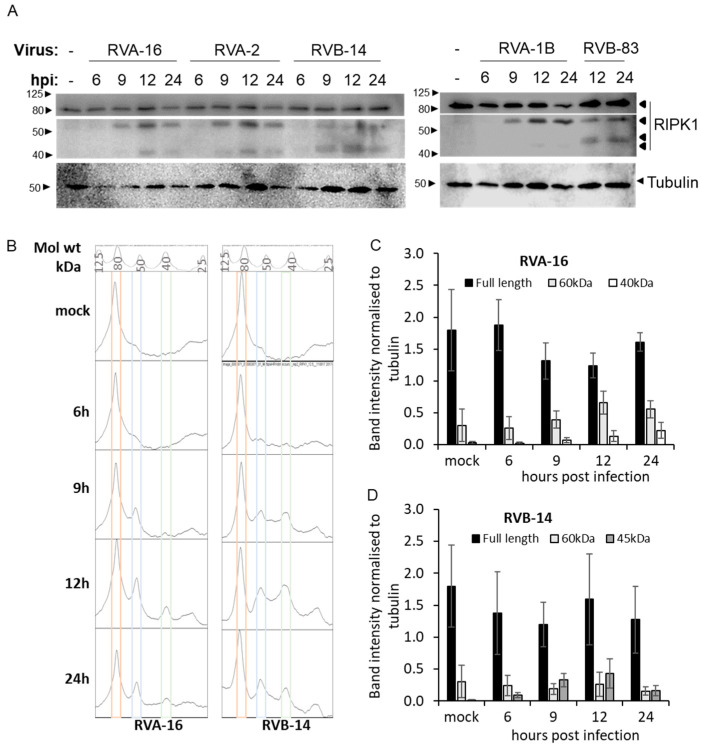
Infection with different RVs results in RIPK1 cleavage. O-HeLa cells were infected with the RV strains indicated at an M.O.I. of 3, for 1 h of adsorption, then cultured for a further 24 h post-infection (hpi). Protein lysates were collected at the indicated times and subject to SDS-PAGE (12.5%), transferred to nitrocellulose membrane and blocked. (**A**) Blots were probed with anti-RIPK1 antibodies. Tubulin was used as a loading control for all blots and is included below the corresponding RIPK1 blots. The position of bands correlating to the indicated full-length protein, or the cleavage products are indicated to the right of the blot. The relevant molecular weights are indicated to the left of the blots. The blots shown are representative of three repeats. (**B**) ImageJ was used to plot the densitometric profile of each of the lanes in the blot; each peak corresponds to a band in the blot. Profile of the molecular weight marker lane and the size corresponding to each peak is shown at the top. Coloured boxes indicate peaks corresponding to RIPK1 bands. (**C**,**D**) Intensity of RIPK1 bands was measured with ImageJ and normalized to the corresponding tubulin band. Data shown are mean ± SEM of three blots.

To determine whether the results that were seen for RVA-16 previously [5] were applicable to other RV strains, we investigated RIPK1 cleavage in infection. After infecting cells for various times within a single round of viral replication (6, 9, or 12 h), lysates were collected and processed for western blot analysis and staining with an antibody to the RIPK1 N-terminal. The 6 and 9 hpi timepoints from RVB-83 infected cells were excluded due to the low growth of the virus, and technical considerations. A ~60 kDa band was detected with the N-terminal RIPK1 antibody in all infection samples from 9 h of infection or more and was not detected in the uninfected control (Figure 1A). Interestingly, in some samples, more than one cleavage product was detected with the anti-RIPK1 antibody, with size differences observed between group A and B viruses. Thus, ~40 kDa product is observed in cells infected with RVA-16, -2, 1B, while a similar product appeared to be closer to ~45 kDa in cells infected with RVB-14, -83. Plotting the profiles of each lane, confirmed this difference, as shown in the representative profiles shown (Figure 1B). Densitometric image analysis for RIPK1 full-length and cleavage products showed a time dependent trend towards reduced full-length protein concomitant with increasing level of 60 kDa cleavage products from 9 hpi.; representative histograms for RVA-16 and RVB-14 are shown in Figure 1C,D respectively. In cells infected with RVA-16, there was a significant increase in 60 kDa product at 12 hpi relative to mock (*p* = 0.007), to 6 hpi (*p* = 0.003) and to 9 hpi (*p* = 0.04), indicative of time dependent increase. The increase in levels of the ~40 kDa band was not statistically significant. In contrast, in cells infected with RVB-14, the increase in levels of the 60 kDa band was not statistically significant while there was a very significant increase in the ~45 kDa band at all times post-infection, relative to mock (*p* < 0.0001).

**Figure 2:**
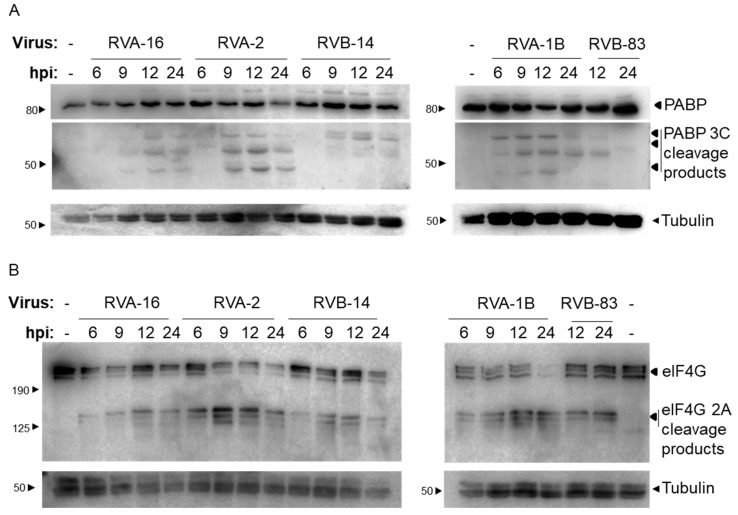
RV protease activity correlates with RIPK1 cleavage. O-HeLa cells were infected with the RV strains as per Figure 1. Protein lysates were collected at the indicated times and subject to SDS-PAGE, transferred to nitrocellulose membrane and blocked. Blots were probed with (**A**) anti-PABP antibodies, or (**B**) anti-eIF4G antibodies. Tubulin was used as a loading control for all blots and is included below the corresponding blots. The position of bands correlating to the indicated full-length protein, or the cleavage products are indicated to the right of the blot. The relevant molecular weights are indicated to the left of the blots. Representative western blots of three independent experiments have been shown. Please note that proteins were separated on 12.5% (**A**) or 8% (**B**) gels.

To confirm that the viral proteases were active at a time correlating with RIPK1 cleavage, two protease cleavage substrates were examined, namely eIF4G and PABP, being substrates of the 2A and 3C proteases, respectively. PABP cleavage products were detected in all infection samples at 9 hpi or later (Figure 2A) and in RVA-1B infected samples at 6 hpi or later. The cleavage profile of PABP differed between cells infected with RVs belonging to species A (RVA-16, RVA-2, RVA-1B) and those infected with RVs belonging to species B (RVB-14 and RVB-83), however, this was not explored further. Additionally, 2A protease activity was confirmed with eIF4G cleavage products detected in all infection samples (Figure 2B). Only the full-length protein (multiple bands, with a major band at ~220 kDa [17,18]) is observed in the non-infected (lanes labelled ‘-’) samples. On infection with RV, cleavage products are observed at ~125 kDa concomitant with reduced full-length protein relative to non-infected samples. We have shown previously [17] that cleavage of eIF4G by RVA-16 2A protease results in multiple cleavage products. The extent and efficacy of the cleavage varied between strains, with a significant reduction of the full-length protein observed at 24 hpi in RVA-2, RVB-14 and RVA-1B infected samples but not RVA-16 or RVB-83 infected samples.

### 3.2. Treatment of Infected Cells with Rupintrivir Prevents RIPK1 Cleavage

**Figure 3:**
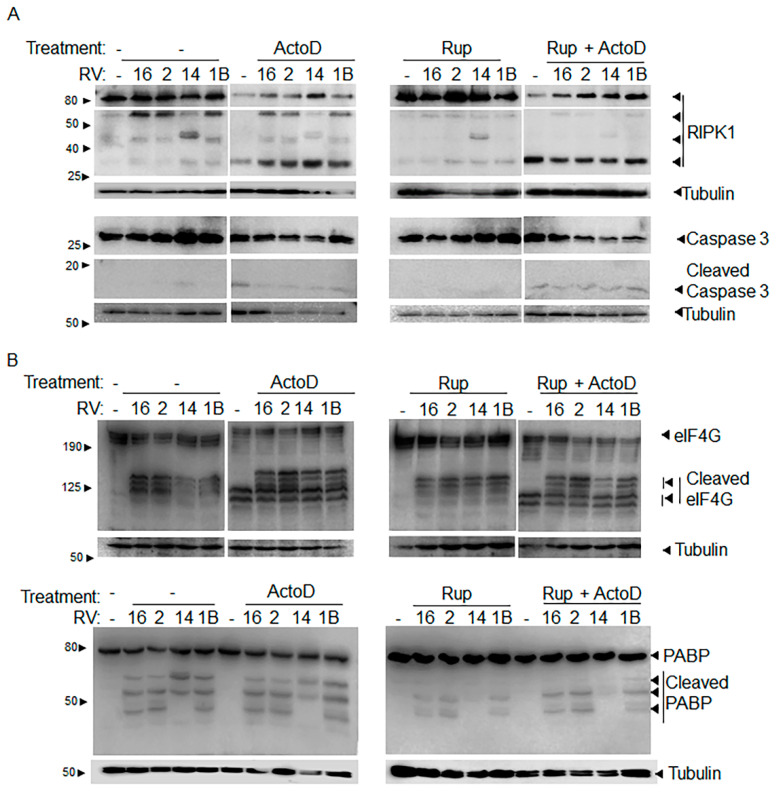
Inhibition of 3Cpro abrogates cleavage of RIPK1. O-HeLa cells were infected with the RV strain indicated at an M.O.I. of 3, for 1 h of adsorption and then a further 12 hpi. At 3 hpi, cells were treated with ActoD (5 µg/mL) or remained untreated, and at 9 hpi, cells were treated with Rupintrivir (1 µM) or remained untreated. Protein lysates were subject to SDS-PAGE, transferred to nitrocellulose membrane and blocked. Blots were probed with (**A**) anti-RIPK1 antibodies (upper blot), anti-caspase 3 antibodies (middle blot), (**B**) anti-eIF4G antibodies (upper blot), anti-PABP antibodies (lower blot). Tubulin was used as a loading control for all blots, and is included below the corresponding blots in (**A**, **B**). The position of bands correlating to the indicated full-length protein, or the cleavage products are indicated to the right of the blot. The relevant molecular weights are indicated to the left of the blots. Representative western blots of three independent experiments have been shown. Please note that proteins were separated on 12.5% (RIPK1, PABP blots) or 8% (eIF4G blot) gels.

Having shown previously that 3Cpro is the major mediator of RIPK1 cleavage in RVA-16 infection [5], we sought to confirm whether this was consistent amongst other RV strains. ActoD treatment induces caspase 8 activity and was used to control for caspase 8 mediated cleavage of RIPK1. To determine whether 3Cpro was responsible for the viral cleavage of RIPK1 we included the specific 3Cpro inhibitor, Rupintrivir (Rup). As expected, ~60 kDa RIPK1 cleavage product was detected in all infected, untreated samples (Figure 3A). Treatment of infected cells with Rupintrivir abrogated the generation of the ~60 kDa RIPK1 cleavage product in infected samples (Figure 3A, compare lanes labelled Rup with lanes with no treatment ‘-’). Interestingly, the ~45 kDa cleavage product observed in RVB-14 infection (and RVB-83 infection, refer to Figure 1) was also present in samples treated with Rupintrivir, thus, likely not a result of 3Cpro activity.

Treatment of infected cells with ActoD appeared to enhance the presence of the RIPK1 cleavage product at ~30 kDa, consistent with caspase 8 activation [5]. The ActoD generated RIPK1 cleavage product was distinct from that seen in infection alone samples, showing that RIPK1 cleavage products detected in infected samples were not due to an apoptotic response to virus infection. That ActoD induced apoptosis was confirmed with detection of caspase 3 cleavage in all ActoD treated samples, with or without infection or Rupintrivir (Figure 3A, cleaved caspase 3). The apoptotic caspase 3 cleavage product was also detected in RV-only infected samples, albeit at a lower level, suggesting that some level of apoptosis is induced in RV infected cells as shown previously [5,7]. This is also confirmed by our analysis of cell death using Flow Cytometry (see below). RV 2A protease activity was confirmed by the detection of eIF4G cleavage products in all infection samples (Figure 3B), regardless of any treatment, confirming that Rupintrivir does not inhibit 2A protease activity. As expected, ActoD treatment also resulted in eIF4G cleavage, although with a pattern different from that in RV infected cells. Rupintrivir activity was confirmed by examining PABP cleavage products. The high molecular weight PABP cleavage product was detected in infection samples, but only when cells were not treated with Rupintrivir (Figure 3B).

### 3.3. Apoptosis Induction by ActoD Inhibits RVs of Species A, but Not Species B

Having established that a key apoptotic adaptor protein is cleaved by 3Cpro of all RV strains tested, we wanted to test whether inducing apoptosis would have an effect on viral replication. The putative 3Cpro cleavage site in RIPK1 is postulated to subvert apoptotic signalling; consistent with this, induction of apoptosis reduces the infectious titre in RVA-16 infected cells [5,7].

**Figure 4:**
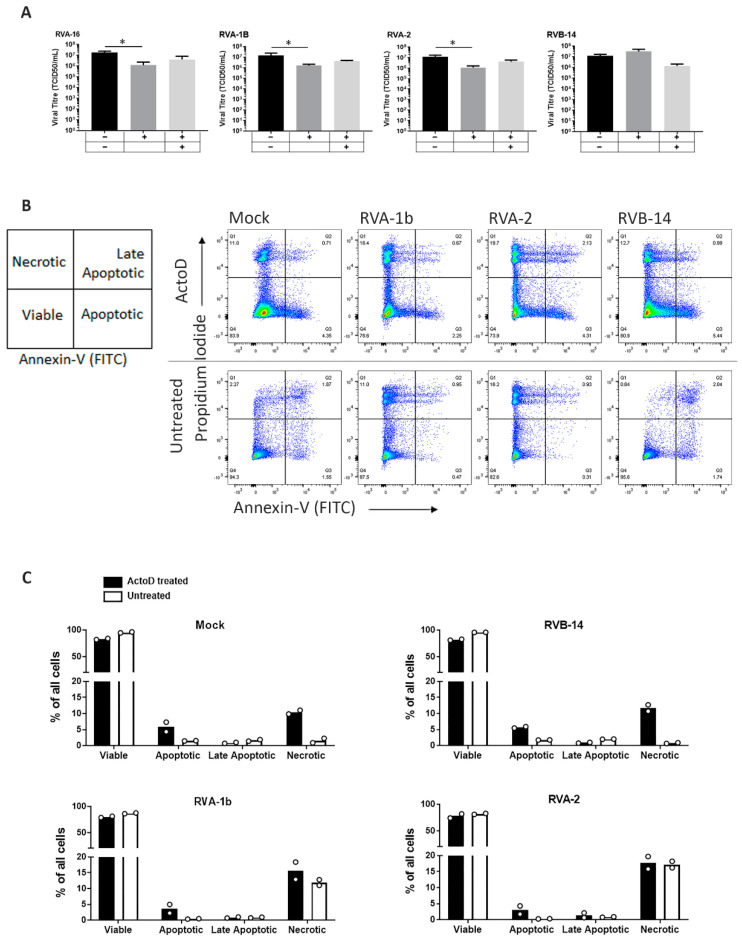
RVA but not RVB strains modulate apoptotic pathways. O-HeLa cells were infected with the RV strain indicated at an M.O.I. of 3, for 1 h of adsorption. (**A**) Cells were treated at 3 hpi with ActoD (5 µg/mL) or remained untreated followed by caspase 8 inhibitor (4 µM) at 4 hpi, or remained untreated. At 24 hpi, virus cultures were frozen and thawed. The virus was clarified and titrated. Results are expressed as the mean of 3 independent experiments and error bars are the SEM. * *p* < 0.05. (**B**) At 3 hpi, cells were treated with ActoD (5 µg/mL) or remained untreated. Both adherent and non-adherent cells were collected at 12 hpi and stained with Annexin-V (FITC) antibodies and Propidium Iodide and processed for FACS analysis. Live cells were subject to doublet discrimination before quadratic gating to determine viable (AnV − Pi−), apoptotic (AnV + Pi−), late apoptotic (AnV + Pi+), or necrotic (AnV − Pi+) populations. Representative FACS plots are shown. (**C**) Quantitation as % of single cells (all cells) of data represented in (**B**). Data are representative of two experiments performed in triplicate and columns represent the mean with the two data points shown on each.

After infecting cells with each of the RV strains, we treated them with ActoD and then with or without caspase 8 inhibitor. Caspase 8 inhibitor was included to test whether any effect on RV replication could be mitigated against in an apoptosis-specific manner. Similar to our previous findings in RVA-16 ([5] and histogram in Figure 4A), apoptosis induction by ActoD significantly reduced the titre of RVA-2 and RVA-1B (*p* < 0.05) and the effect was partially reversed with caspase-8 inhibition (Figure 4A). Contrary to these results, neither ActoD nor caspase 8 inhibition had any effect on RVB-14, in keeping with previous data showing that RVB-14 infection benefits from apoptosis induction [3].

### 3.4. Subgroup A, but Not Subgroup B, Strains Modulate Apoptosis

To characterise the cell death induced in RV infected cells, flow cytometry of infected cells was conducted to analyse Propidium iodide (Pi) incorporation (necrosis) or phosphatidylserine externalization (apoptosis). The percentage of cells (after doublet discrimination) positive for Annexin V (An), or Pi, or both, was plotted. A set of representative flow cytometry scattergrams is shown in Figure 4B, with associated histograms (mean ± standard error from two independent experiments) shown in Figure 4C.

RVA-1b and RVA-2, but not RVB-14, caused necrosis and modulated apoptosis (Figure 4B compare scattergrams; Figure 4C, compare clear columns) when compared with non-infected control (scattergram and histogram labelled ‘mock’). RVB-14 infection had no effect on ActoD induced cell death (Figure 4C, compare filled columns in ‘mock’ with ‘RVB-14′). In presence of RVA-1b or RVA-2 infection, ActoD induced higher apoptosis than infection alone (Figure 4C, compare filled columns with clear columns ‘RVA-1b’ and ‘RVA-2′) and this was not different from that induced in the mock infected cells (compare filled columns for ‘Apoptotic’ data in ‘RVA-1b’ or ‘RVA-2′ with ‘mock’). ActoD did not cause any necrosis over and above that caused by the infection (compare filled and clear columns for ‘Necrotic’ data in ‘RVA-2′ and ‘RVA-1B’). Clearly, RVA-1B/-2 (minor group) modulate apoptotic and necrotic pathways, however, this is dissimilar to the modulation by RVA-16 (major group) [5,7], which does not induce necrosis.

## 4. Discussion

There are over 150 RV strains, thus while we have previously shown a mechanism by which RVA-16 may limit cell death and promote viral replication [5], it is important to understand whether this is a conserved mechanism amongst RV strains. In the current study, we show that RIPK1 is cleaved by the 3Cpro of each of the representative RV strains tested. In doing so, we have provided the first comparative study on RVs and the effects of apoptosis induction. We chose representative RVs with either close or distant phylogenetic relationships (species A or B), or similar or dissimilar receptor usage (minor or major groups). By doing so, we have shown that the cleavage of RIPK1 is not only conserved amongst all tested strains, but the cleavage profile of the protein is similar between RVs from the same species. This conservation of RIPK1 cleavage points to it being an important virus-host cell interaction.

The treatment of infected cells with Rupintrivir, a 3Cpro specific inhibitor [19,20], allowed us to eliminate the possibility of other viral proteases cleaving RIPK1. As we had previously described for RVA-16 [5], one prominent cleavage product was evident without 3Cpro inhibition and not detected with 3Cpro inhibition in RVA-1b and RVA-2. Surprisingly, we found that even with 3Cpro inhibition, RIPK1 degradation products were observed in cells infected with RVB-14 correlating with work from other groups showing that 2A protease substrate specificity and cleavage kinetics differs between species [21]. Examination of the 2A protease from A and B-species RVs showed significant differences in the orientation of key amino acids within the enzyme from each species of RV, and this differential folding likely contributes to substrate specificity [22]. Additionally, the native cleavage site between VP1 and 2A within the viral polyprotein differs between RVA-2 and RVB-14 [22,23,24]. These previous papers agree with our findings, that in addition to conserved 3Cpro mediated cleavage of RIPK1, RVB-group viruses may also cleave RIPK1 via the 2A protease. Closer examination of our data (compare Figure 1A and Figure 3A) the 60 kDa cleavage product of 3Cpro activity does not increase over time, while the ~45 kDa cleavage product of 2Apro activity increases over time. Our data suggest initial 3Cpro activity in RVB-14 infected cells that does not appear to continue; however, this needs to be confirmed. The putative cleavage of RIPK1 by the RVB 2A protease resulted in a RIPK1 fragment of ~45 kDa, and this was exclusive from (caspase 8-mediated) fragments seen with apoptosis induction. Within RIPK1, cleavage at Tyrosine 387 by the B-group RV 2A protease, aligns with the predicted RVB 2A N-terminal RIPK1 cleavage product of ~43 kDa. While we have not definitively confirmed the 2A cleavage site within RIPK1, it should be noted that Y-G constitutes the same P_1_ and P’ amino acids as the native RVB-14 2A protease cleavage site (within the polyprotein) [25].

As stated, the treatment of cells with Rupintrivir reduced the known RIPK1 3Cpro cleavage product [5] in cells infected with any virus tested. The sequences of 3Cpro from RVs of different RV species do not have strict sequence identity, however, the catalytic triad (His 40, Glu 71, Cys 147) has absolute conservation [26]. Importantly, high conservation also extends throughout the substrate binding groove [26], which, unlike the 2A protease, may dictate low substrate divergence. Further to the highly consistent nature by which RIPK1 is cleaved by RV 3Cpro, the poliovirus 3C protease also results in a similar RIPK1 cleavage product [27]. The cleavage of RIPK1 by the RV 3Cpro occurs at a position that may separate key adaptor and signalling domains, thus is proposed to subvert apoptotic signalling [5,7]. The death domain (DD) of RIPK1, which is downstream of the putative 3Cpro mediated cleavage site, interacts with other proteins that contain a DD, such as TRADD and FADD and have cell-death inducing functions [28]. Additionally, the RHIM domain, which is essential for the formation of RIPK1/3 dimers and necroptosis induction [29,30], is also downstream of the proposed cleavage site. The cleavage of RIPK1 may subvert apoptosis specifically [7], however, the means by which the cells proceed through programmed, or infection-induced cell death remains inconclusive.

We investigated the means of cell death induced in our cell culture by various RVs. We have previously demonstrated that RVA-16 infection does not result in detectable, late-stage apoptosis [5], again in line with previous studies of both RV [7] and other picornaviruses [1]. We aimed to extend our previous work, and the work of others (cited above), and analyse cell-death pathways involved in RV infection, in a comparative manner. We found that RVA-2 and RVA-1B infection resulted in necrotic cell death whereas RVB-14 did not. Interestingly, treatment with an apoptosis inducer reduced RVA-2 and RVA-1B titre but not RVB-14 titre. Together, these results point to complex interactions between cell death pathways and virus infections. Inducing apoptosis may reduce the titre of A species RVs, in which cell death may normally proceed through an apoptotic-independent mechanism, as seen by the level of necrotic cell death in the Annexin V/Pi assay. RVB-14 infection has previously been shown to benefit from apoptosis induction [3], in line with our data, whereby viral titre was not reduced with apoptosis induction.

## 5. Conclusions

In conclusion, we have extended our previous findings to show that the cleavage of RIPK1 occurs in infection by many strains of RV, and 3Cpro is the mediator in all cases. Additionally, the 2A protease from RVs belonging to genotype B may also cleave RIPK1. We hypothesize that the cleavage of RIPK1 may be mechanistically involved in the suppression of caspase-dependent cell death and promote a necrotic phenotype in cells infected with RVA viruses. Despite species divergence, the control of cell death remains an essential component of establishing viral infection and subsequent pathogenesis.

## Data Availability

All data associated with this study are presented in the article.
